# Characterisation of Innate Fungal Recognition in the Lung

**DOI:** 10.1371/journal.pone.0035675

**Published:** 2012-04-20

**Authors:** Inês Faro-Trindade, Janet A. Willment, Ann M. Kerrigan, Pierre Redelinghuys, Sabelo Hadebe, Delyth M. Reid, Naren Srinivasan, Helen Wainwright, Dirk M. Lang, Chad Steele, Gordon D. Brown

**Affiliations:** 1 Division of Immunology, Institute of Infectious Disease and Molecular Medicine, University of Cape Town, Observatory, South Africa; 2 Aberdeen Fungal Group, Section of Immunology and Infection, Division of Applied Medicine, Institute of Medical Sciences, Foresterhill, University of Aberdeen, Aberdeen, United Kingdom; 3 Division of Anatomical Pathology, University of Cape Town, Observatory, South Africa; 4 Department of Human Biology, University of Cape Town, Observatory, South Africa; 5 Department of Medicine, University of Alabama, Birmingham, Alabama, United States of America; Montana State University, United States of America

## Abstract

The innate recognition of fungi by leukocytes is mediated by pattern recognition receptors (PRR), such as Dectin-1, and is thought to occur at the cell surface triggering intracellular signalling cascades which lead to the induction of protective host responses. In the lung, this recognition is aided by surfactant which also serves to maintain the balance between inflammation and pulmonary function, although the underlying mechanisms are unknown. Here we have explored pulmonary innate recognition of a variety of fungal particles, including zymosan, *Candida albicans* and *Aspergillus fumigatus*, and demonstrate that opsonisation with surfactant components can limit inflammation by reducing host-cell fungal interactions. However, we found that this opsonisation does not contribute directly to innate fungal recognition and that this process is mediated through non-opsonic PRRs, including Dectin-1. Moreover, we found that pulmonary inflammatory responses to resting *Aspergillus* conidia were initiated by these PRRs in acidified phagolysosomes, following the uptake of fungal particles by leukocytes. Our data therefore provides crucial new insights into the mechanisms by which surfactant can maintain pulmonary function in the face of microbial challenge, and defines the phagolysosome as a novel intracellular compartment involved in the innate sensing of extracellular pathogens in the lung.

## Introduction

The increase in fungal infections over the last few decades, particularly with normally commensal or non-pathogenic fungi, has prompted renewed interest in elucidating the mechanisms of protective host anti-fungal immunity. The identification of innate recognition systems have been a focus in many laboratories, and several non-opsonic fungal pattern recognition receptors (PRRs) have been described, including members of the C-type lectin and Toll-like receptor (TLR) families [1]. Dectin-1, a signalling C-type lectin receptor for β-glucans, has been of particular interest, being a key innate receptor required for the control of several fungal pathogens, including *Aspergillus fumigatus* [2], [3]. Recognition by these PRRs is thought to occur at the cell surface, triggering intracellular signalling events which lead to the initiation of protective immune responses [4]. For Dectin-1, intracellular signalling is critically dependent on the Syk kinase/CARD9 pathway and, in macrophages, additionally requires co-stimulation of MyD88-coupled TLRs for the induction of inflammatory cytokines [2]. However, much less is known about the contribution of the opsonic mechanisms of fungal recognition, particularly those mediated by pulmonary surfactant.

Pulmonary surfactant is a complex mixture of lipids and proteins that are required for respiration, by reducing the surface tension at the alveolar air-liquid interface, and which play an important role in host defence. The host defence functions of surfactant are mediated by several components including, for example, surfactant protein (SP)-A and SP-D, and complement [5], [6]. In general, surfactant opsonisation promotes microbial aggregation and enhanced uptake by alveolar macrophages and other immune cells [7]. This enhancement of uptake is thought to be mediated, at least in part, by interactions of surfactant with opsonic receptors, which in turn modulate the resultant inflammatory responses [6], [8]. However, the level of involvement of the opsonic receptors for surfactant proteins in microbial recognition is still unclear, as are the mechanisms by which pulmonary inflammatory responses to infection are controlled *in vivo*. Here we have examined the opsonic and non-opsonic mechanisms involved in the innate recognition of various fungal particles in the lung.

## Results

### The effect of surfactant opsonisation on fungal recognition

**Figure 1:**
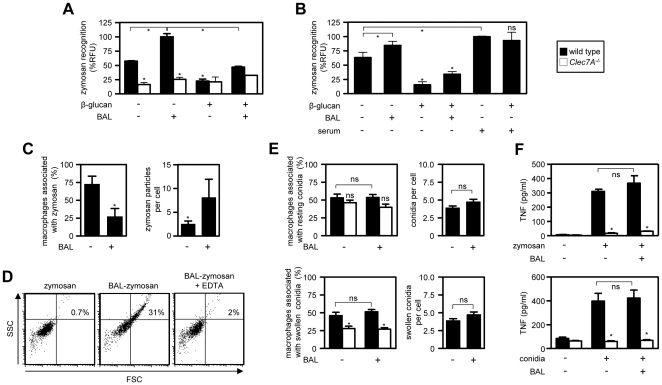
Surfactant-mediated aggregation modulates the number of particle-cell contacts but does not influence non-opsonic recognition. (A) Binding of unopsonised or surfactant-opsonised (BAL) zymosan to alveolar macrophages isolated from wild-type (black bars) or Dectin-1-deficient (white bars) mice, in the presence or absence of soluble β-glucan. Total fluorescence is represented as a percentage of fluorescent units (RFU), relative to the surfactant-opsonised zymosan sample. (B) Binding of surfactant-opsonised zymosan or unopsonised particles to peritoneal macrophages isolated from wild-type mice in the presence or absence of soluble β-glucans, as indicated. Recognition of serum opsonised zymosan is included for comparison. Total fluorescence is represented as a percentage of relative fluorescent units (RFU), normalized to the serum-opsonised sample. (C) Quantitation of binding of unopsonised or surfactant-opsonised zymosan to alveolar macrophages by microscopy. (D) FSC and SSC flow cytometric analysis of zymosan, BAL-opsonised zymosan, and BAL-opsonised zymosan in the presence of 100 mM EDTA. (E) Quantitation of the effects of surfactant opsonisation on the binding of *A. fumigatus* resting or swollen conidia, as indicated, to alveolar macrophages isolated from wild-type mice (black bars) or Dectin-1-deficient (white bars) mice by microscopy. (F) TNF production from alveolar macrophages isolated from wild-type (black bar) or Dectin-1-deficient (white bars) mice following the addition of unopsonised or surfactant-opsonised zymosan or resting *A. fumigatus* conidia, as indicated. (A)–(D) The data shown are the mean+SD and are representative of at least two independent experiments. (E)–(F) The data shown are mean+SEM of data pooled from two independent experiments. *p<0.05.

We first examined the effect of surfactant on the recognition of zymosan, a model fungal particle derived from the cell wall of *Saccharomyces cerevisiae* [9], by alveolar macrophages *in vitro*. To include all of the components of surfactant that are normally involved during fungal opsonisation in the lung, we used unpurified murine bronchoalveolar lavage (BAL)-fluid for our experiments. We observed that treatment of zymosan with BAL-fluid (which we term “BAL-opsonization”) resulted in a modest, but significant, increase in binding of these particles to alveolar macrophages (ranging from 1.2–1.8 fold), when total binding by the cellular population was determined by fluorometry (**Figure 1A**; black bars). This was consistent with observations for other microbial particles, following their opsonisation with purified surfactant proteins [7], and similar results were obtained with thioglycollate-elicited peritoneal macrophages, which are also used frequently to study the effects of surfactant on microbial recognition (**Figure 1B**).

When analysed more closely, however, looking at each cell individually, we observed that BAL-opsonisation resulted in a significant decrease in the actual number of cells within the total population that were associated with zymosan (**Figure 1C** and **Figure S1A & B**). However, in those cells that had bound zymosan, there was a substantial increase in the number of particles per cell (**Figure 1C** **and Figure S1A & B**). By flow cytometry and microscopy, we could demonstrate that this increased per-cell uptake was due to particle aggregation, whose formation was directly induced following BAL-opsonisation and could be inhibited with EDTA (**Figure 1D** and **Figures S1B**), and which resulted in the deposition of SP-D on the zymosan surface (**Figure S1C**).

We have previously demonstrated that the recognition of unopsonised zymosan by leukocytes is mediated by Dectin-1, and that this interaction can be specifically inhibited by the addition of soluble β-glucans [9]. As opsonic receptors for surfactant constituents are thought to contribute to particle binding [8], [10], we next examined the mechanisms of recognition of BAL-opsonised zymosan. To inhibit Dectin-1, we pre-incubated alveolar macrophages with β-glucan, and measured the amount of opsonised zymosan binding by fluorometry (**Figure 1A**; black bars). We observed that the binding of BAL-opsonised zymosan was inhibited by the addition of soluble β-glucans, to a level comparable to the inhibition obtained with unopsonised zymosan. Similar results were also observed using thioglycollate-elicited peritoneal macrophages (**Figure 1B**). Furthermore, as a control in this experiment, we included serum-opsonised zymosan whose binding to macrophages was not inhibitable by β-glucan, indicating that the recognition of these particles was now occurring through opsonic receptors for complement and other serum opsonins (**Figure 1B**).

To exclude the possibility of interference by the soluble β-glucan inhibitors themselves [11], [12], we also examined particle recognition in alveolar macrophages deficient in Dectin-1 (**Figure 1A**; white bars). As expected [13], the binding of unopsonised zymosan was largely abrogated in these cells, moreover, the binding of BAL-opsonised zymosan was similarly reduced. Thus the binding of BAL-opsonised zymosan does not involve additional recognition through opsonic receptors for constituents of isolated lung surfactant.

We next explored the role of pulmonary surfactant in the innate recognition of *Aspergillus fumigatus*, a well characterised pulmonary pathogen [14], whose opsonisation with purified surfactant proteins induces conidial aggregation [15]. However, we found that BAL-opsonisation of *A. fumigatus* conidia did not induce significant particle aggregation (**Figure S1D**). The recognition of *Aspergillus* is mediated by several non-opsonic receptors, including Dectin-1, although recognition by this receptor is restricted to swollen and germinating conidial forms which have exposed β-glucans [16]. Dectin-1 has been suggested to be involved in binding resting conidia [17], which lack exposed β-glucans, but we observed no difference between wild type and Dectin-1 deficient cells, either in the number of cells associated with resting conidia or in the number of resting conidia per cell (**Figure 1E**; upper panels). This indicates that Dectin-1 is not involved in the recognition/binding of *A. fumigatus* resting conidia. In contrast, and consistent with the stage-specific exposure of β-glucans [16], we observed a significant Dectin-1 dependence for the binding of swollen conidia (**Figure 1E**; lower panels). Notably, BAL-opsonisation had no effect on the recognition of either of these particles by alveolar macrophages.

Proteins within surfactant can influence inflammatory responses; an activity that is proposed to depend, at least in part, on interactions with their cellular receptors [6], [8], [10], [18]. We therefore examined the effects of BAL-opsonisation on the inflammatory cytokine responses of alveolar and thioglycollate-elicited macrophages by measuring the production of TNF in response to BAL-opsonised and unopsonised zymosan and *Aspergillus* resting conidia (**Figure 1F** and **Figure S1E**). As we have shown previously [3], [9], the inflammatory cytokine response to both types of unopsonised fungal particle was Dectin-1-dependent, and could be abrogated by the use of cells deficient in this receptor. Importantly, we observed that the inflammatory cytokine responses to both particles remained Dectin-1-dependent following BAL-opsonization. Thus, collectively, these data show that the binding and TNF responses to surfactant-opsonised fungi occurs through non-opsonic mechanisms.

### Aggregation by surfactant limits pulmonary inflammatory pathology

Our *in vitro* data indicated that microbial aggregation mediated by BAL-opsonisation reduces the total number of interactions with host cells, and was suggestive of a mechanism by which pulmonary surfactant could facilitate anti-microbial immunity while simultaneously reducing the distribution of pulmonary inflammation. This model would therefore predict that inhibition of surfactant function upon challenge with particles that usually become aggregated, such as zymosan, would result in significantly increased particle distribution within the lung and exacerbated inflammatory responses. Conversely, blockage of surfactant function with particles which do not become aggregated, such as *Aspergillus* conidia, should have no effect on distribution or inflammation.

**Figure 2:**
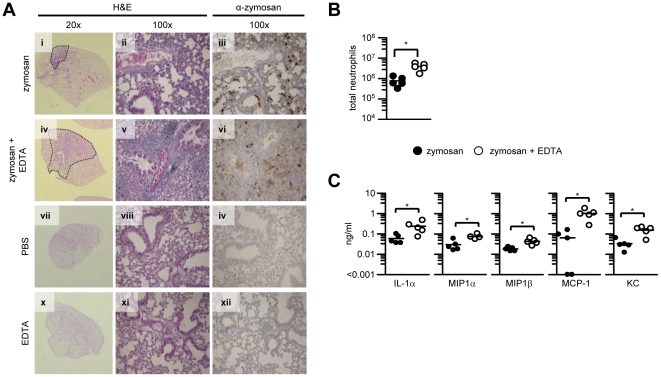
Inhibition of surfactant function exacerbates pulmonary inflammation in response to zymosan particles. (A) Pulmonary histology following intra-tracheal administration of zymosan, zymosan plus EDTA, PBS or EDTA, stained with haematoxylin and eosin (H&E) or for the presence of zymosan (α-zymosan), as indicated. (B) Quantitation of neutrophil (CD11b^+^GR-1^+^) cells in the lungs of infected mice. (C) Production of selected cytokines and chemokines in the lungs of individual mice following intra-tracheal challenge with zymosan or zymosan plus EDTA. The data shown are representative of at least two independent experiments, except for (B) which is a single experiment. *p<0.05.

To demonstrate this experimentally, we examined pulmonary inflammation following the intratracheal administration of zymosan particles with or without EDTA (**Figure 2**). We chose to use EDTA to block surfactant function, as this approach would allow for the transient but simultaneous inhibition of the surfactant proteins as well as the other contributing components, such as complement. Furthermore, EDTA has minimal effects on pulmonary function and inflammatory responses, being often used in nebulizer solutions in humans [19], and we did not detect any significant effects when administered intratracheally to mice (see **Figure 2A** and **Figure S3**, discussed below).

The intratracheal administration of zymosan induced a localised peribronchial and perivascular inflammation with limited numbers of inflammatory cells in the alveolar spaces (**Figure 2A** panels i and ii). In contrast, the co-administration of zymosan with EDTA, induced marked widespread peribronchial and perivascular inflammation with significant inflammatory infiltrates in the alveolar spaces (**Figure 2A** panels iv and v). Consistent with a lack of aggregation, and correlating with the pulmonary inflammation, the addition of EDTA resulted in a diffuse distribution of the zymosan particles (**Figure 2A** panel vi), compared to the discrete aggregates of zymosan observed in the absence of EDTA (**Figure 2A** panel iii). Flow cytometric and microscopic analysis of BAL-cells, isolated following i.t. administration of FITC-labelled zymosan, confirmed that EDTA treatment reduced zymosan aggregation as well as the number of particles per cell *in vivo* (**Figure S2A & B**). In line with our histological observations, we found significant quantitative increases in neutrophil recruitment in the lungs of mice receiving zymosan plus EDTA, compared to those administered with zymosan alone (**Figure 2B**), which correlated with substantially increased inflammatory and neutrophil-chemoattractant cytokines (**Figure 2C**). Animals receiving PBS or EDTA alone showed no signs of inflammation (**Figure 2A** panels vii to xi).

To next wanted to confirm that our observations with zymosan would also reflect intact fungal particles which can become aggregated following BAL-opsonisation. We therefore examined the effect of EDTA during pulmonary challenge with *Candida albicans*, an atypical pulmonary pathogen [20] which has previously been used to study surfactant function [21]. We could demonstrate *in vitro* that live *C. albicans* yeast particles became aggregated following BAL-opsonisation (**Figure S2C**). Importantly, as we found for zymosan, the co-administration of EDTA with *C. albicans* intratracheally to mice resulted in exacerbated inflammatory responses compared to animals administered with yeast particles alone (**Figure S2D**).

**Figure 3:**
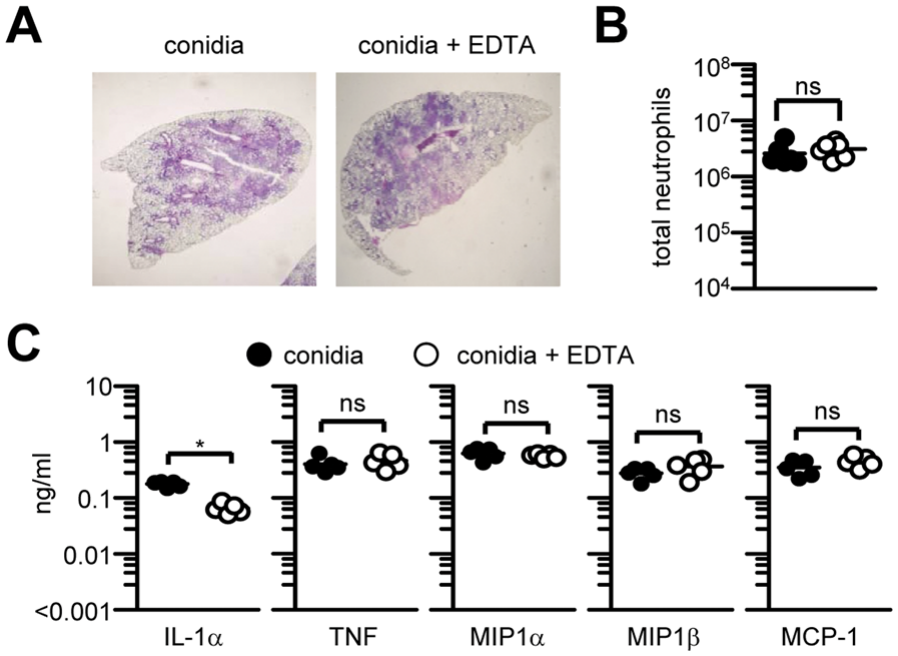
Inhibition of surfactant function has no effect on pulmonary inflammation in response to *A. fumigatus* resting conidia. (A) Haematoxylin and eosin staining of pulmonary sections following intra-tracheal administration of *A. fumigatus* resting conidia in the presence or absence of EDTA. (B) Quantitation of neutrophil (CD11b^+^GR-1^+^) cells in the lungs of infected mice. (C) Production of selected cytokines and chemokines in the lungs of individual mice after intra-tracheal challenge with resting conidia or resting conidia plus EDTA. The data shown are representative of at least two independent experiments. *p<0.05.

We next examined pulmonary pathology following the intratracheal administration with *A. fumigatus* resting conidia, which are similar in size to zymosan (∼3 µm), in the presence or absence of EDTA and found equivalent levels of peribronchial inflammation distributed throughout the lung in both groups, as determined by histological analysis (**Figure 3A**). Importantly, the distribution of inflammation within the lung of the Aspergillus treated mice was similar to that induced by the same number of zymosan particles in the presence of EDTA. Furthermore, and consistent with the histological observations, we found no difference in neutrophil recruitment (**Figure 3B**) nor in inflammatory cytokines and chemokines (**Figure 3C**) in the lungs of mice challenged with *Aspergillus* conidia in the presence of EDTA, compared to those challenged with conidia alone. Thus, the transient inhibition of surfactant function by EDTA had no effect on early pulmonary inflammation following challenge with *A. fumigatus* conidia. Overall, these data suggest that fungal particle aggregation mediated by surfactant helps to limit particle dissemination throughout the lung and the extent of early pulmonary inflammation.

### The role of Dectin-1 in the recognition of *Aspergillus* and zymosan in vivo

**Figure 4:**
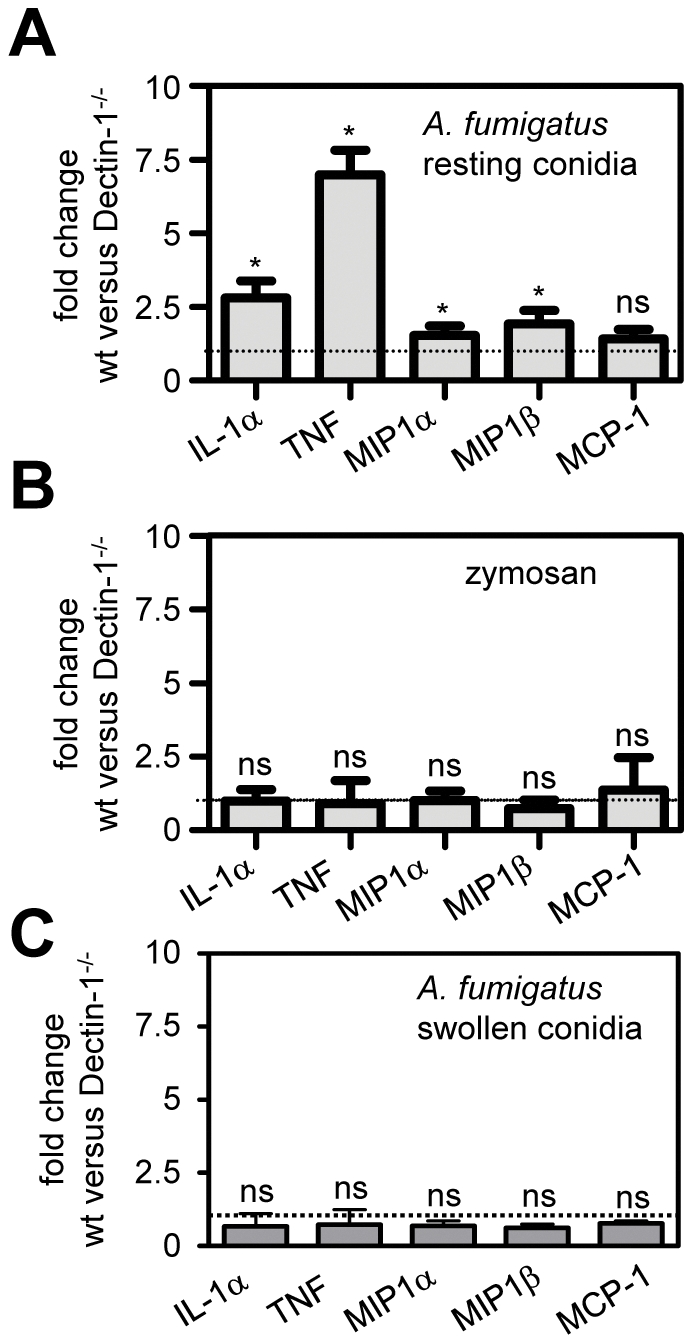
Dectin-1 is only required for inflammatory responses to *Aspergillus* resting conidia *in vivo*. (A) The Dectin-1 dependency of selected pulmonary cytokines and chemokines expressed as the fold change of wild type responses versus those obtained in Dectin-1 deficient mice, following the intra-tracheal administration of resting *A. fumigatus* conidia in the presence of EDTA. (B) The Dectin-1 dependency of selected pulmonary cytokines and chemokines expressed as the fold change of wild type responses versus those obtained in Dectin-1 deficient mice, following the intra-tracheal administration of zymosan in the presence of EDTA. (C) The Dectin-1 dependency of selected pulmonary cytokines and chemokines expressed as the fold change of wild type responses versus those obtained in Dectin-1 deficient mice, following the intra-tracheal administration of *A. fumigatus* swollen conidia. The data shown are the mean+SD of relative protein levels, determined by cytokine ELISA, and are representative of at least two independent experiments, except for (C) which is data from two pooled independent experiments. *p<0.05.

Given the importance of non-opsonic recognition through Dectin-1 in the *in vitro* response to both BAL-opsonised and non-opsonised fungal particles (see **Figure 1**), we next examined pulmonary inflammation in Dectin-1 deficient animals in the presence or absence of EDTA to inhibit surfactant function. We first looked at *Aspergillus*, where Dectin-1 has been shown to play a central role in the induction of inflammatory responses and control of this pathogen *in vivo* [3]. Consistent with these observations, we found that infection of Dectin-1-deficient animals with *A. fumigatus* resting conidia resulted in significant impairment in the production of numerous cytokines and chemokines, in comparison to wild-type animals. As before, the inhibition of surfactant function with EDTA had no effect, even in the Dectin-1 deficient animals (**Figure 4A**). In contrast, and despite the convincing *in vitro* data (see **Figure 1**), we found that Dectin-1 was not essential for inflammatory responses to zymosan *in vivo*, even in the presence of EDTA (**Figure 4B**); a finding similar to that reported previously [22].

Zymosan can be recognised by several cell surface PRRs [23] and receptors such as Dectin-2 [24] are likely to be compensating in the absence of Dectin-1 *in vivo*; an area we are currently exploring. However, *Aspergillus* possesses similar PAMPS to zymosan and is recognised through the same PRRs [14], so the *in vivo* Dectin-1 requirement for the recognition of this organism, but not zymosan, was puzzling. Unlike zymosan, however, resting conidia are immunologically inert [25], and inflammatory responses to conidia are only induced once their PAMPS become exposed, following conidial swelling and germination [16], [26]. This suggested that the masking of PAMPs on resting conidia was somehow conferring the Dectin-1 dependency. To confirm this hypothesis, we infected mice with *A. fumigatus* swollen conidia and observed that the inflammatory response to these particles was now Dectin-1-independent (**Figure 4C**). Furthermore, and consistent with our prior observations, the addition of EDTA had little effect on these early inflammatory responses (**Figure S3**). Importantly, this result demonstrates that EDTA does not affect the PRR systems involved in the detection of PAMP-exposed particles *in vivo*. Therefore, these data demonstrate that innate recognition of inhaled fungal particles is not influenced by surfactant opsonisation and involves differential utilization of Dectin-1. Notably, Dectin-1 was only essential for the *in vivo* recognition of resting *Aspergillus* conidia which, paradoxically, lack exposed β-glucan.

### The innate recognition of *Aspergillus* occurs intracellularly

**Figure 5:**
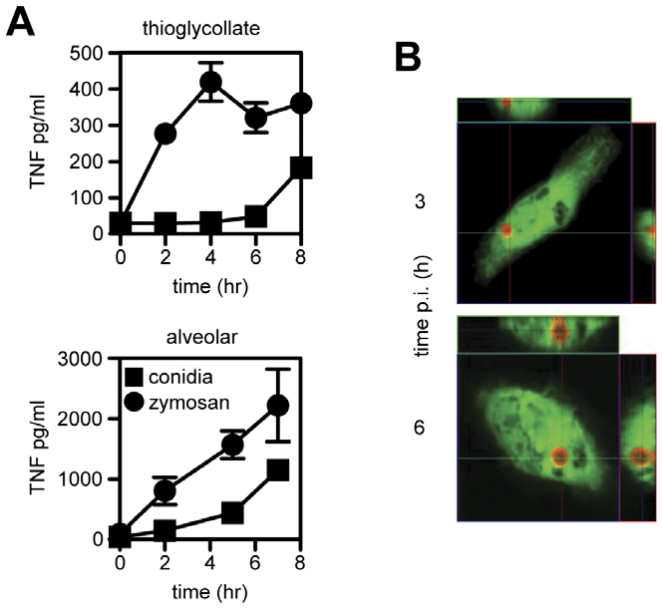
The induction of inflammatory responses to *Aspergillus* resting conidia is delayed and occurs after particle uptake. (A) The production of TNF over time following the infection of thioglycollate-elicited or alveolar macrophages with zymosan particles (black circles) or resting *Aspergillus* conidia (black squares), as indicated. The data shown are the mean ± SD, and are representative of at least two independent experiments. *p<0.05. (B) Orthogonal confocal projections of CFSE-labelled thioglycollate-elicited macrophages (green) 3 and 6 hr after the uptake of resting *Aspergillus* conidia (labelled with calcofluor, red).

Resting conidia are rapidly ingested by macrophages following inhalation into the lung [27], suggesting that the innate recognition of these particles by Dectin-1 *in vivo* may be occurring following their uptake by macrophages. To explore this possibility, non-opsonised conidia or zymosan particles were added to alveolar or thioglycollate-elicited peritoneal macrophages for one hour, the cells washed to remove unbound particles, and the inflammatory responses assessed over time by measuring the production of TNF (**Figure 5A**). Consistent with recognition at the cell surface [28], there was an immediate and robust induction of TNF following the addition of zymosan. Similar results were observed following the stimulation of macrophages with *Aspergillus* swollen conidia, which have exposed β-glucan ([16]; **Figure S4A**). In contrast, Dectin-1-dependent inflammatory responses to resting *Aspergillus* conidia were significantly delayed and occurred only several hours after infection, corresponding to the emergence of swollen conidial forms [27] (**Figure 5A** and **Figure S4B**). By confocal analysis, we could demonstrate that all the resting conidia were fully internalised by 3 hr after infection and that they had swollen by 6 hr (**Figure 5B**), but were still located within these vesicles, when the induction of the inflammatory response was evident. This suggested that the induction of inflammatory responses to this organism were only occurring after the conidia had been internalised.

**Figure 6:**
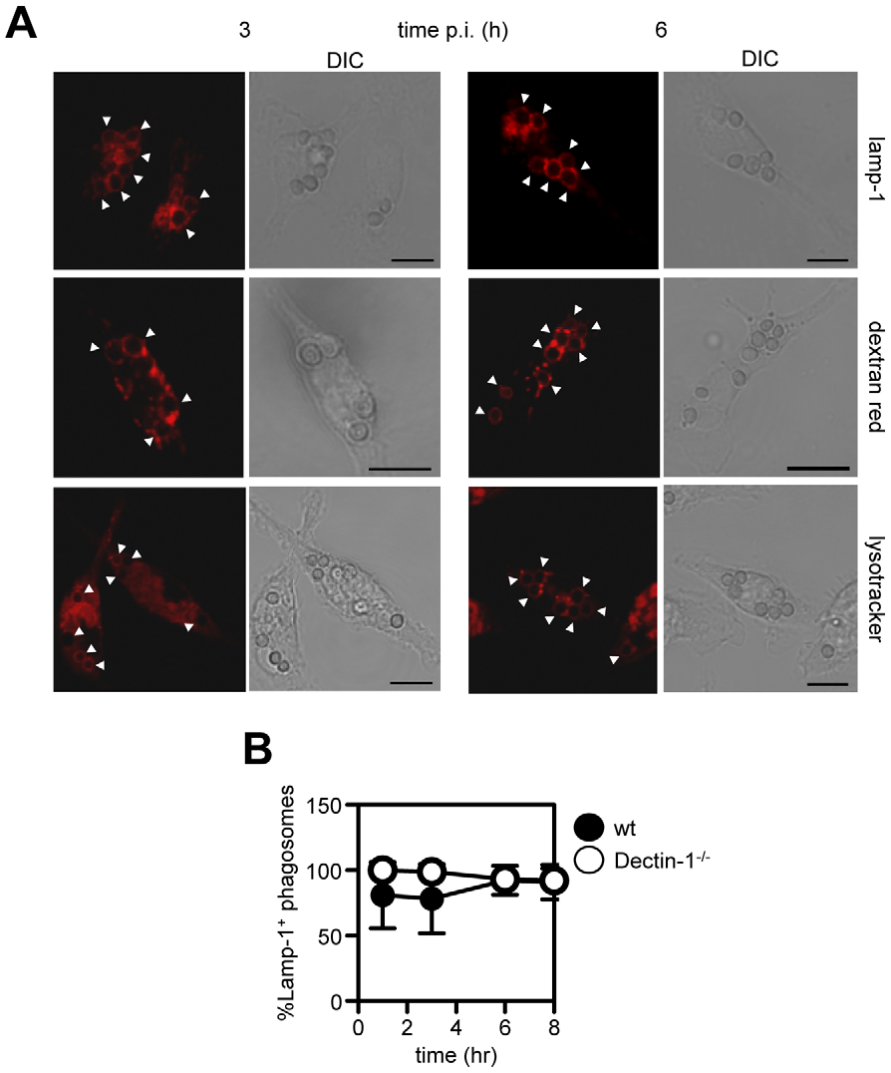
Phagosomes containing *Aspergillus* resting conidia mature normally into phagolysosomes. (A) Immunofluorescent confocal analysis of *Aspergillus* conidia phagosomes in thioglycollate-elicited macrophages at 3 and 6 hr after infection, and examined for acquisition of LAMP-1, preloaded Dextran-red and fluorescence of lysotracker, indicating acidification. Arrowheads point to the location of conidia. Scale bar indicates 10 µm. (B) Kinetic analysis, determined by immunofluorescence microscopy, of the acquisition of Lamp-1 to conidial phagosomes in thioglycollate-elicited macrophages from wild type (black circles) and Dectin-1^−/−^ (white circles) mice. Data shown are mean ± SD. The data shown are representative of at least two independent experiments.

We next investigated the nature of the phagosomes containing the internalised conidia in thioglycollate-elicited peritoneal macrophages (for ease of isolation and imaging), looking at two time points corresponding to the period prior to (3 hr), and after the start of (6 hr), TNF production. We first examined the acquisition of the late endosomal/lysosomal marker Lamp1 (lysosomal membrane protein 1), and observed that conidial phagosomes had acquired this marker at both time points (**Figure 6A**). Indeed, this marker was acquired within 1 hr following conidal uptake and was retained by the phagosomes for all subsequent time points measured (**Figure 6B**). We then examined phagosome maturation by pre-labelling lysosomes with the fluid-phase marker Dextran-Texas Red and looked for acquisition of this fluorescent marker in the conidial phagosomes. At three hours, conidial phagosomes were observed to co-localize with Dextran-Texas red and this co-localization was also evident at the later time point (**Figure 6A**). Acidification is a well defined marker of mature phagosomes, so the pH of this organelle was next examined with a membrane diffusible dye, LysoTracker DND-99, which becomes trapped in acidic compartments. Acidification of conidial phagosomes was observed at 3 hr post-infection, as previously described [29], [30], but all phagosomes were acidified by 6 hr, when the inflammatory response was initiated (**Figure 6A**). Acidification could be inhibited with bafilomycin A, indicating that phagosomal acidification was being mediated by the vacuolar ATPase (**Figure S5**). These data therefore demonstrate that by 3 hr, vesicles containing *Aspergillus* conidia have matured normally and have developed into phagolysosomes.

Dectin-1 can influence the intracellular fate of β-glucan bearing ligands [9], and we examined the possibility that this receptor was involved in phagosomal maturation, as has been suggested for other PRRs, including the TLRs [31]. However, analysis of Dectin-1^−/−^ macrophages did not reveal any significant alteration of Lamp-1 association (**Figure 6B**), acquisition of Dextran-Texas red, or acidification of the conidial phagosomes (data not shown), at any of the time points we analysed.

### Innate recognition by Dectin-1 in the phagolysosome

**Figure 7:**
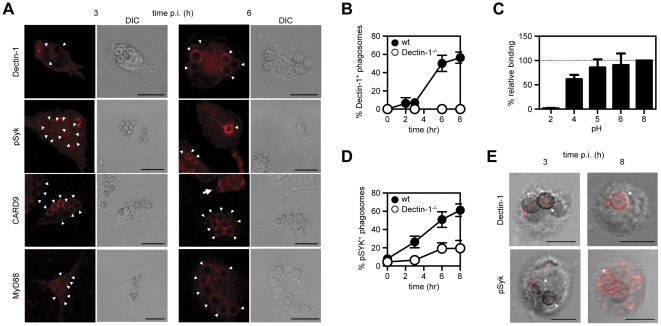
Phagolysosomes containing *Aspergillus* conidia acquire innate signalling components. (A) Immunofluorescent confocal analysis of phagosomes containing *Aspergillus* conidia at 3 and 6 hr after infection of thioglycollate-elicited macrophages, showing the recruitment of Dectin-1, phosphoSyk, CARD9 or MyD88. Scale bar indicates 10 µm. Arrowheads point to the location of conidia; bold arrow indicates cytoplasmic straining. (B) Kinetic analysis of the acquisition of Dectin-1 to the conidial phagosomes in wild type and Dectin-1^−/−^ thioglycollate-elicited macrophages. (C) Flow cytometric analysis of the influence of pH on the ability of soluble Fc-Dectin-1 to bind to zymosan. (D) Kinetic analysis of the acquisition of phosphoSyk to conidial phagosome in wild type and Dectin-1^−/−^ thioglycollate-elicited macrophages. (E) Immunofluorescent confocal analysis of BAL-cell phagosomes 3 and 8 hr after i.t. infection of mice with resting Aspergillus conidia, showing the recruitment of Dectin-1 and phosphoSyk (pSyk) to the conidial phagosome *in vivo*. Scale bar indicates 10 µm. Arrowheads point to the location of conidia. The data shown are representative of at least two independent experiments.

The recognition of fungal particles such as zymosan and *Candida* by Dectin-1, and induction of inflammatory cytokines, has previously been shown to occur at the cell surface, whereas we and others have previously shown that internalization results in the loss of this receptor from the phagosomal membrane and termination of these responses [9], [28], [32]. In contrast, here we observed that the Dectin-1 dependent response to *Aspergillus* conidia occurred several hours after uptake (see **Figure 5A**), when the particles were located in acidified phagolysosomes. To understand how Dectin-1 could be contributing to the recognition of resting *Aspergillus* conidia following their uptake, we examined the cellular distribution of this receptor in thioglycollate-elicited macrophages at 3 hr and 6 hr after infection, as described above. By 3 hr, Dectin-1 appeared to be membrane located, as expected [9], and was not observed to associate with the conidial phagosomes (**Figure 7A**). However, 6 hr after infection, following conidial swelling and exposure of β-glucans [27], there was distinct association of Dectin-1 with the conidial phagolysosomes. A time course analysis revealed that the kinetics of this association (**Figure 7B**) mirrored that observed for the induction of TNF (see also **Figure 5A**). Although the ligand binding ability of many receptors can be influenced by phagosomal pH, Dectin-1 was still able to interact with fungal particles at low pHs, such as those found in phagolysosomes [33] (**Figure 7C**). Thus the innate recognition of ingested *Aspergillus* resting conidia is being mediated by Dectin-1 in acidified phagolysosomes following conidial swelling.

Signalling for TNF production via Dectin-1 in macrophages occurs through Syk kinase [34], and we could demonstrate that this pathway was being activated in acidified phagolysosomes by immunostaining for phosphorylated Syk (p-Syk) during conidial uptake (**Figure 7A**). Although a low level of association of p-Syk was detected on the conidial phagosomes at all time points, even in Dectin-1-deficient macrophages, there was robust recruitment of this kinase on conidial phagosomes by 6 hr, corresponding to the induction of the inflammatory response (**Figure 7A** and **Figure 7D**).

Notably, we could also demonstrate this phenomenon *in vivo*; that there was an association of Dectin-1 and pSyk with conidial phagolysosomes and that this only took place several hours following i.t. administration of resting conidia to mice (**Figure 7E**). Furthermore, cellular uptake of conidial aggregates were not observed in any of the BAL isolates, supporting our prior *in vitro* observations that BAL- opsonisation of these particles does not result in aggregation (compare **Figure S2B** with **Figure 7E**).

Signalling downstream of Syk occurs, in part [35], through the adaptor CARD9 [36], which was shown to be recruited to early phagosomes expressing Dectin-1 during the uptake of zymosan [37]. Indeed, we observed that CARD9, which is normally cytoplasmically associated (**Figure 7A**, bold arrow [37]) was also recruited to phagosomes containing *Aspergillus* conidia. However, unlike zymosan phagosomes [37], CARD9 remained localised at the phagosomal membrane at both the early and later time points, suggesting that recruitment of this adaptor to fungal phagosomes was independent of Dectin-1 localisation (**Figure 7A**).

In addition to the Syk/CARD9 pathway, the induction of TNF in macrophages by Dectin-1 requires collaborative signalling through MyD88-coupled TLRs [9], [34], and loss of MyD88 significantly reduces macrophage inflammatory responses to *Aspergillus* conidia [26]. Consistent with these observations, we observed that MyD88 was also present at the phagosomal membrane at both time points (**Figure 7A**). Overall, our data therefore demonstrates that the requirement for Dectin-1 in the innate response to *Aspergillus* resting conidia *in vivo*, stems from the recognition of this pathogen in acidified phagolysosomes.

## Discussion

The opsonisation of microbes with pulmonary surfactant modulates their uptake and the resultant inflammatory response; effects which are thought to occur, at least in part, through interactions with opsonic receptors for surfactant on phagocytes [5]. Although underlying mechanisms behind these activities of surfactant are still incompletely understood, it is thought to play an essential role in maintaining the balance between pulmonary function and the generation of protective anti-microbial responses to inhaled microorganisms. Here we have explored the contribution of surfactant opsonisation in the innate recognition of various fungal particles and have found that surfactant opsonisation makes no direct contribution to particle recognition or early inflammatory responses. While we observed that zymosan became aggregated following opsonisation with BAL fluid, we could show using inhibitors and receptor knockout mice that the recognition of these aggregates was still mediated through non-opsonic mechanisms. Given that the innate recognition of zymosan by macrophages is a model system, and arguably one of the best understood interactions [9], [38], [39], it is possible that the recognition of other microbes following surfactant opsonisation [7] is similarly being mediated through non-opsonic PRRs. Furthermore, opsonisation with surfactant may mask the non-opsonic ligands on certain organisms, which could explain the decreased binding and responses that have been described following opsonisation of pathogens, such as *Mycobacterium tuberculosis*, *Blastomyces dermatitidis* and *Pneumocystis carinii* [7].

The reduction in the number of host cells coming into contact with fungal particles, following the aggregation caused by BAL-opsonisation, suggested a mechanism by which surfactant opsonisation could limit pulmonary inflammation. Indeed, we could demonstrate this experimentally by transiently blocking surfactant function with EDTA, which led to enhanced pulmonary inflammation with both zymosan and *C. albicans*, which also becomes aggregated following surfactant opsonisation. In contrast, the addition of EDTA during the administration of *Aspergillus* conidia, which do not become aggregated following surfactant opsonisation, had little effect on pulmonary inflammation. Furthermore, the inflammation induced by *Aspergillus* resting conidia was distributed throughout the lung in a manner similar to that observed when zymosan aggregation was inhibited by EDTA. The influence of EDTA on the inflammatory responses to zymosan, and *Candida*, were not due to effects on the PRR systems involved in their recognition (see below), as we could demonstrate that the inflammatory responses induced by *Aspergillus* swollen conidia remained unaffected by this treatment. However, as with any inhibitor, we cannot fully exclude off-target effects. Our data therefore indicate that the ability of surfactant to balance pulmonary function versus inflammation, following the inhalation of microbes, is achieved initially by reducing host-cell contacts through microbial aggregation.

Although difficult to compare directly, our results are somewhat at odds with the current concept of a more direct role for the surfactant proteins (such as SP-A and SP-D; largely used in purified form) in microbial recognition and modulation of host responses [5], [40]. However, we feel that the validity of our findings stem from the two unbiased approaches we undertook in our experiments. Firstly, for our *in vitro* studies, we used unpurified bronchoalveolar lavage fluid to opsonise our particles, instead of individually purified surfactant proteins. Although purified surfactant proteins have been widely used to characterise the host protective functions of surfactant, the use of these proteins in isolation does not take into account their combinatorial effects, especially in light of their functional differences, or the contributions of the other surfactant components, such as lipids, which are known to influence these interactions [40]. Furthermore, in some cases, purified surfactant proteins have been contaminated with immunmodulators, including cytokines such as TGF-β, influencing experimental outcomes [5], [41].

The second important and unbiased approach we used for these experiments was the use of EDTA to block the interactions of surfactant *in vivo*. We chose this route as it allowed for the transient, but simultaneous, inhibition of the major surfactant proteins and other components, and also avoided the complications of alterations in homeostasis that are observed in the surfactant protein deficient animals [5], [10]. While only transiently inhibiting surfactant function, and therefore excluding any interpretation of the longer term effects of surfactant on inflammation (see below), this approach provided *in vivo* experimental evidence that following the inhalation of microbes, the primary function of surfactant is to aggregate these particles and limit the amount of contact with host cells. These results may also provide a possible explanation, at least in part, for the enhanced microbial dissemination and exacerbated inflammatory responses that have been observed in surfactant protein deficient animals, following infection with various microbial pathogens [7], [42].

Our data also differ from previous studies looking at the role of surfactant proteins during infections with *Aspergillus*. *In vitro*, surfactant proteins have been reported to cause conidial aggregation and enhanced phagocytosis by leukocytes [11], [15]; effects which we did not observe either *in vitro* (**Figure S1D**) or *in vivo* (**Figure 7E**). Importantly, the former experiments were performed using purified proteins and not intact surfactant, whose other constituents can influence these interactions, as discussed above. Indeed, the hydrophobic components of surfactant have been shown to inhibit the interactions between surfactant proteins and *Aspergillus* conidia [43].

Previous studies using gene-deficient mouse models or the administration of purified proteins to infected mice have also suggested that surfactant proteins play significant roles during *Aspergillus* infection *in vivo* [18], [44]. However, these experiments were all performed in immune-suppressed animals and, in the case of the knockout animals, were further complicated by the alterations in lung homeostasis discussed above. Thus our data are not directly comparable to these previous observations. Never the less, our results suggest that in the absence of aggregation, surfactant plays only a minor role during the initial pulmonary responses to *Aspergillus* conidia. It is important to note, however, that our observations only reflect the early innate response to inhaled conidia and do not exclude a role for surfactant during the later stages of infection.

As surfactant was not involved in modulating innate fungal recognition, we explored the non-opsonic pulmonary mechanisms involved in fungal sensing; focussing particularly on the beta-glucan receptor Dectin-1. Intriguingly, pulmonary responses to fungal particles with exposed beta-glucans, including swollen *Aspergillus* conidia and zymosan, were not Dectin-1 dependent *in vivo*, despite the strict requirement for this receptor *in vitro* ([16], [38] and this study). These data therefore suggest that there are other innate fungal recognition systems in the lung, which have yet to be defined, but may involve more recently identified receptors such as Dectin-2 [24] or Mincle [45]. A role for complement, TLR2, TLR4 and MyD88 has already been ruled out [22].

In contrast to these PAMP-exposing particles, we found that recognition of *Aspergillus* resting conidia was strictly Dectin-1 dependent *in vivo*, suggesting an alternative mechanism of innate recognition. The recognition of intact extracellular pathogens is thought to occur at the cell surface or in early endosomes [4], and the interaction of Dectin-1 with fungal particles does occur at these cellular locations [16], [32]. Indeed, detailed characterization of the phagocytosis of zymosan and *Candida* has shown that Dectin-1 associates with fungal particles at the cell surface, and in early phagosomes, but then dissociates from these compartments shortly after particle uptake [32]. Although the Dectin-1 mediated recognition of *Aspergillus* has been shown to occur at the cell surface, and in early phagosomes, this required prior swelling of the conidia and exposure of β-glucans [16], [26]. Here we could demonstrate both *in vitro* and *in vivo* (**Figure 7**) that Dectin-1 mediated recognition of *Aspergillus* resting conidia in the lung took place intracellularly, in phagolysosomes, following the phagocytosis and swelling of these particles. While it is still unclear how this receptor is recruited to the phagolysosome, and whether it is recycled from the cell membrane or originates from *de novo* synthesis, this intracellular mechanism of recognition explains why inflammatory responses to *Aspergillus* resting conidia were strictly Dectin-1 dependent *in vivo*.

Previously thought to be a degradative compartment whose primary function is killing of ingested pathogens and the generation of microbial peptides for presentation to lymphocytes [33], our data demonstrates that the phagolysosome is also a site for the innate detection of extracellular pathogens. Furthermore, this is the first example of recognition being mediated by a member of the C-type lectin family in these compartments. While nucleic acids, presumably released following microbial degradation, are known to be detected in lysosomes [46], [47], [48], our findings also show that intact organisms and other microbial components are sensed in these intracellular vesicles. Indeed the phagolysosome may be a central compartment for innate recognition of ingested microbes; an attractive hypothesis, considering the many, normally masked, PAMPs which become exposed or released during the maturation of these vesicles [49], [50].

In summary, our data has provided novel insights into the mechanisms by which surfactant modulates pulmonary pathology and our exploration of how these components influence non-opsonic fungal recognition has revealed that the phagolysosome is an important intracellular organelle for the innate sensing of extracellular pathogens.

## Materials and Methods

All animal experimentation conformed to animal care and welfare protocols approved by the Ethical Review Committee and Faculty of Heath Science Animal Ethics Committee of the Universities of Aberdeen (project license number 60/4007) and Cape Town (license number: 09/055), respectively, and in strict accordance with the guidelines for the usage of animal in laboratory research of the South African Association for Laboratory Animal Science and the UK home office.

### Mice and isolation of primary murine macrophages

Alveolar macrophages and bronchoalveolar lavage (BAL)-fluid were isolated from 6- to 8-week old female 129SvEv wild-type or 129SvEv Dectin-1 knockout mice [13], or BALB/c mice, using standard methodology [51]. In brief, the lungs were lavaged with 1 ml of cold PBS, followed by 10 washes with PBS containing 0.1% EDTA [52]. The first, PBS-only, washes from all mice were pooled, the cells recovered by centrifugation, and the corresponding supernatants (representing the BAL) were harvested. Cells recovered from all lavages, usually >98% enriched for alveolar macrophages, were pooled and washed in RPMI medium (RPMI 1640 plus 10% v/v heat-inactivated foetal calf serum, 10 mM L-glutamine, and 10 mM sodium pyruvate). The isolation of thioglycollate-elicited macrophages, was performed as described [53]. All cells were plated in triplicate the day before use in 48-well plates at 5×10^4^ or 1×10^5^ cell per well for binding or cytokine assays, respectively.

### *In vitro* particle binding and stimulation assays

To prepare the zymosan particles, unlabeled (Sigma-Aldrich) or FITC-labelled (Molecular Probes) zymosan particles were sonicated to obtain a single particle suspension and were then opsonised in BAL-fluid diluted 1∶1 with RPMI for 1 hr at room temperature on a rotating wheel. The ability of BAL-fluid to induce comparable levels of zymosan aggregation (see **Figure 1D**) was used as an internal control in all experiments (data not shown). Unopsonised particles were treated similarly, except RPMI medium instead of BAL-fluid was added. Zymosan aggregation was determined by flow cytometric analysis and by microscopy. Similar procedures were used to generate surfactant-opsonised resting or swollen *A. fumigatus* (isolate 13073; ATCC, Manassas, VA) conidia, prepared as described previously [16], and *C. albicans* (SC5314), grown in yeast form as described [54].

For the binding and stimulation assays, macrophages were washed in RPMI medium at room temperature and fungal particles (10–20 per cell) were added. When required, soluble β-glucans (100 µg/ml) were added 20 min prior to, and kept throughout the assay. The cells were then incubated for 40 min at room temperature, to allow the particles to settle, and then for 30 min (zymosan) or 60 min (*Aspergillus*) at 37°C. All wells were then thoroughly washed with RPMI medium to remove unbound particles, and the cells directly analysed for zymosan or conidial binding or incubated for a further 3 hr (zymosan; [38]) or 24 hr (*Aspergillus*;[16]) at 37°C, to measure cytokine production. Zymosan binding was quantified either by flow cytometery, fluorescent microscopy or as total fluorescence, while binding of *A. fumigatus* conidia was quantified by microscopy.

For microscopy, cells were plated on glass-coverslips and fluorescent images of zymosan-FITC particles, and/or phase contrast images of cells were collected using a conventional fluorescent microscope (Zeiss Axiovert 40). Image quantification was performed on >100 cells for each different condition. For total fluorescence measurements, the cells were lysed in 3% Triton X-100 and FITC-zymosan in lysates was quantified on a Fluoroskan Ascent FL (Thermo). For cytokine measurements, TNF secretion in the supernatants was assayed by ELISA (Becton Dickinson, Mountain View, CA).

### Confocal microscopy

Macrophages were seeded in 24-well plates at 2×10^5^ cells per well on glass-coverslips in RPMI medium and cultured at 37°C overnight. The cells were washed, infected with conidia (3–20 per cell), and then cultured for 1 hr at 37°C. Unbound conidia were then removed by washing thoroughly, and the cells incubated at 37°C for the times indicated. Fluid phase labelling of lysosomes with Dextran-Texas Red (MW 10 000 Da) and detection of phagosomal acidification using lysotracker Red DND-99 (Molecular Probes) was performed as described previously [55]. Antigens in fixed and permeabilised cells were detected with the following antibodies: 7G7 (anti-Dectin-1 [32]), phosphoSYK (Cell Signalling), LAMP-1 (clone 1D4B, developmental studies hybridoma bank, Iowa), MyD88 (Santa Cruz Biotechnology) and CARD9 (Santa Cruz Biotechnology). Cells were analysed by confocal laser scanning microscopy on a Zeiss LSM 510 META or Zeiss LSM710 confocal microscope, and the settings used for image acquisition were established using cells stained with secondary antibody only controls (data not shown). Images were processed using Adobe Photoshop. For quantification, between 70 and 100 cells were scored for each condition and time point. Where required, conidia were stained with calcofluor and cell membranes with CFSE.

### Effects of pH

Soluble Fc-Dectin-1 [56] (10 µg/ml) was added to zymosan particles and incubated at 4°C for 1 hr. Particles were then washed three times at the various pHs, normalized to pH 7, and the remaining Fc-Dectin-1 bound to these particles was detected with PE-labelled anti-human Fc (Jackson Immunoresearch Laboratories) and analysed by flow cytometry.

### *In vivo* challenges

1×10^7^ unlabelled zymosan and *A. fumigatus* resting conidia, or 1×10^5^ *C. albicans*, in a volume of 50 µl, in the presence or absence of EDTA (100 mM), was administered to the caudal oropharynx of anesthetized mice. Unless otherwise indicated, mice were sacrificed and the lungs were excised at 24 hr (*Aspergillus*, *Candida*) or 72 hr (zymosan) post-inoculation. The times chosen represented the peak inflammatory response to each particle (data not shown). One half of the lung was homogenised and analysed for cytokine and chemokine levels using the Bio-Plex Protein Array System (Bio-Rad, Hercules, California, United States), as per manufacturer's instructions. The remainder of the lung was fixed and processed for histology by staining with haematoxylin and eosin, or with anti-zymosan antibodies (Molecular Probes; 10 µg/ml), which were detected with anti-rabbit HRP (Dako). For quantitative measurements of inflammation as well isolation of cells for *ex-vivo* confocal microscopy, inflammatory cells from the lungs of infected animals were isolated by repeated lavage using PBS with 5 mM EDTA. Neutrophilic components (GR-1^+^, CD11b^+^) were determined by flow cytometry, as described [53]. At least five mice per group were used in all experiments, and all experiments were repeated at least once, unless otherwise indicated.

## Supporting Information

Figure S1 **Surfactant-mediated aggregation of zymosan, but not Aspergillus, modulates the number of particle-cell contacts, but does not affect inflammatory responses.** (A) Representative fluorescent images of alveolar macrophages with fluorescent zymosan or surfactant-opsonised (BAL)-zymosan. (B) Dot plots and quantitative data, determined by flow cytometry, of the interaction of BAL-opsonised and unopsonised FITC-labelled zymosan with thioglycollate elicited macrophages *in vitro*. The effect of EDTA on uptake is also shown. MFI, mean fluorescence intensity. (C) Demonstration of the deposition of SP-D on BAL-opsonised (clear histogram), but not unopsonised (grey histogram), zymosan as determined by flow cytometery. (D) FSC and SSC flow cytometric analysis of unopsonised and BAL-opsonised Aspergillus resting conidia. (E) Peritoneal macrophages from wild-type mice produce comparable amounts of TNF when they bind unopsonized or surfactant-opsonized zymosan particles, and this response can be inhibited by the addition of soluble β-glucan. Data shown are mean ± SEM of data pooled from two independent experiments. *p<0.05.
(TIF)

Figure S2 **Surfactant mediated aggregation of zymosan occurs** ***in vivo*** **and is important for controlling inflammatory responses to C. albicans.** (A) Flow cytometric quantitation of the uptake of FITC-labelled zymosan by phagocytes *in vivo*, in the presence of absence of EDTA. MFI, mean fluorescence intensity (B) Representative fluorescent images of BAL-cells showing the uptake of FITC-labelled zymosan particles (green) in the presence or absence of EDTA. Nuclei are stained with DAPI (blue). (C) FSC and SSC flow cytometric analysis of unopsonised and BAL-opsonised *C. albicans* yeast. (D) Quantitation of neutrophil (CD11b^+^GR-1^+^) cells in the lungs of individual mice following intra-tracheal challenge with *C. albicans* in the presence or absence of EDTA.
(TIF)

Figure S3 **Surfactant does not influence pulmonary inflammatory response to Aspergillus swollen conidia.** Production of selected cytokines and chemokines in the lungs of individual mice following intra-tracheal challenge with Aspergillus in the presence or absence of EDTA.
(TIF)

Figure S4 **The induction of inflammatory responses to** ***Aspergillus*** **resting conidia is delayed and is Dectin-1 dependent.** (A) The production of TNF at 4 and 8 hr following the infection of thioglycollate-elicited macrophages from wild-type mice with various particles, including swollen Aspergillus conidia, as indicated. Unstimulated cells are shown as a control. (B) The production of TNF over time following the infection of thioglycollate-elicited macrophages with resting *Aspergillus* conidia in wild-type or Dectin-1^−/−^ mice, as indicated. The data shown are the mean ± SD, the wild type data is the same as shown in **Figure 5**.
(TIF)

Figure S5 **Acidification of conidial phagosomes can be inhibited by bafilomycin A1.** Immunofluorescent confocal analysis of lysotracker red-labelled phagosomes at 6 hr after infection with and without bafilomycin A. Scale bar indicates 10 µm.
(TIF)
