# Supplementary figures and images for: Characterisation of Innate Fungal Recognition in the Lung
Source: PLoS One. 2012 Apr 20;7(4):e35675. doi: 10.1371/journal.pone.0035675 (PMC3334970; doi:10.1371/journal.pone.0035675)

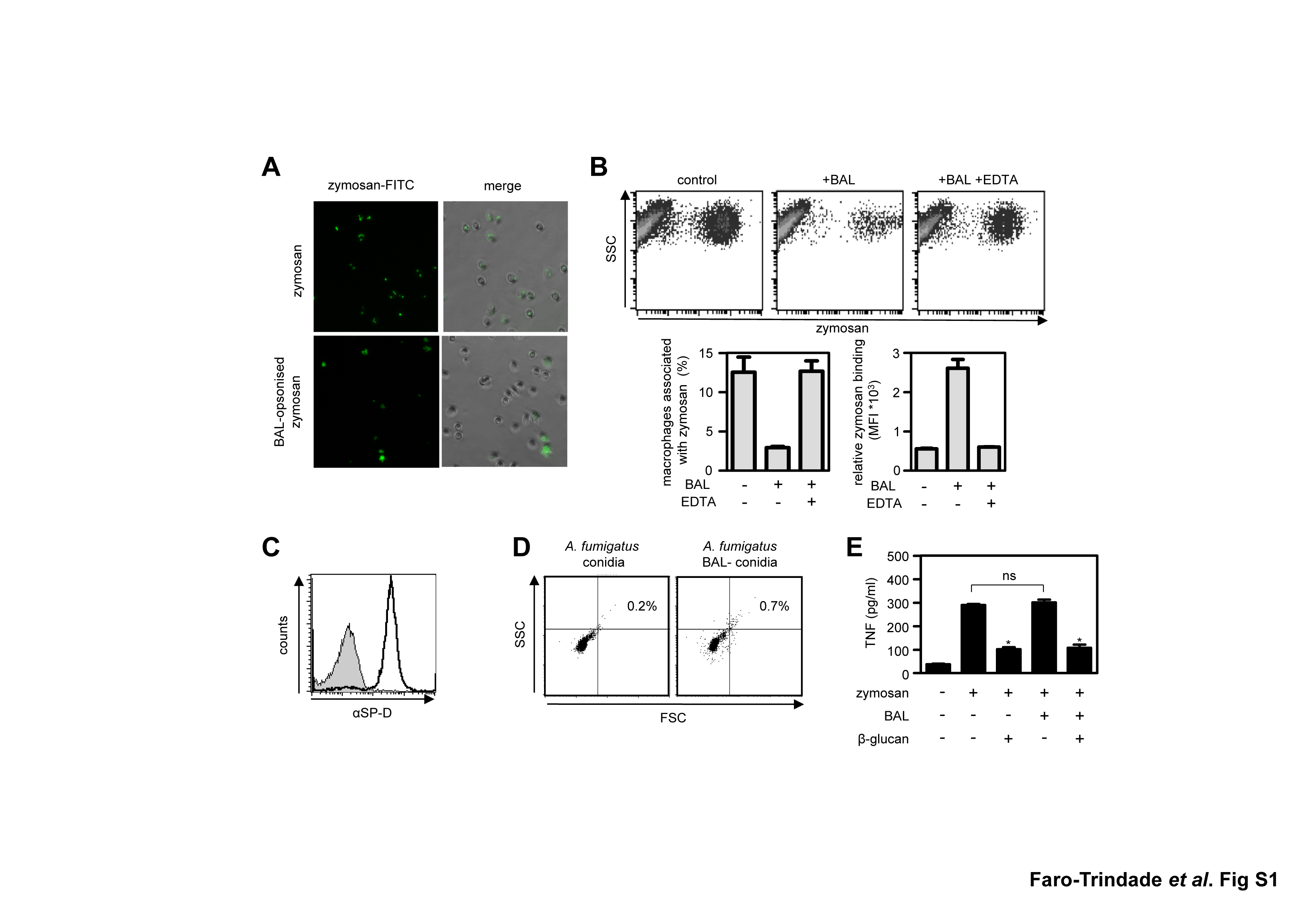

Supplement: Figure S1 — Surfactant-mediated aggregation of zymosan, but not Aspergillus, modulates the number of particle-cell contacts, but does not affect inflammatory responses. (A) Representative fluorescent images of alveolar macrophages with fluorescent zymosan or surfactant-opsonised (BAL)-zymosan. (B) Dot plots and quantitative data, determined by flow cytometry, of the interaction of BAL-opsonised and unopsonised FITC-labelled zymosan with thioglycollate elicited macrophages in vitro. The effect of EDTA on uptake is also shown. MFI, mean fluorescence intensity. (C) Demonstration of the deposition of SP-D on BAL-opsonised (clear histogram), but not unopsonised (grey histogram), zymosan as determined by flow cytometery. (D) FSC and SSC flow cytometric analysis of unopsonised and BAL-opsonised Aspergillus resting conidia. (E) Peritoneal macrophages from wild-type mice produce comparable amounts of TNF when they bind unopsonized or surfactant-opsonized zymosan particles, and this response can be inhibited by the addition of soluble β-glucan. Data shown are mean ± SEM of data pooled from two independent experiments. *p<0.05. (TIF) [file pone.0035675.s001.tif]

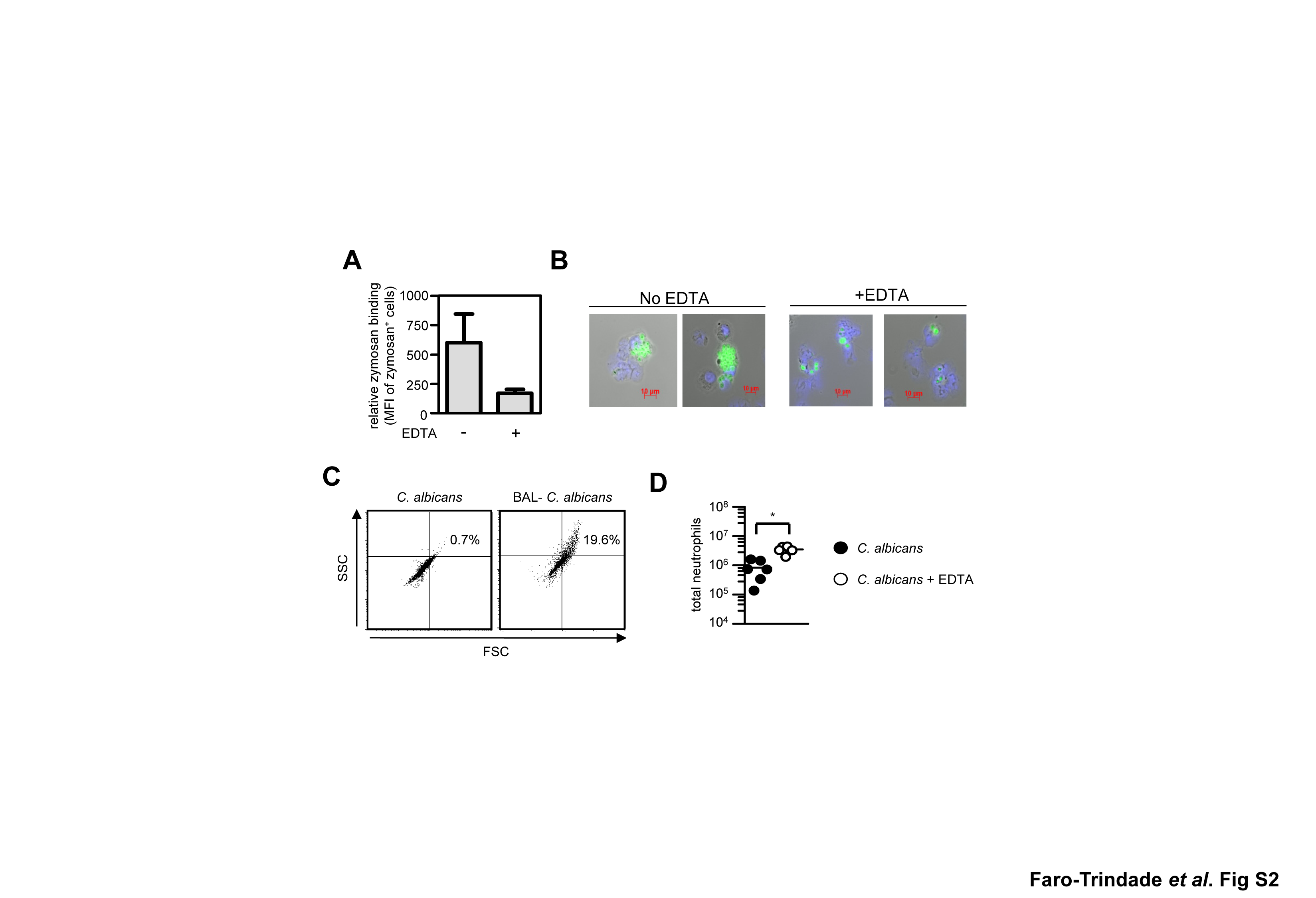

Supplement: Figure S2 — Surfactant mediated aggregation of zymosan occurs in vivo and is important for controlling inflammatory responses to C. albicans. (A) Flow cytometric quantitation of the uptake of FITC-labelled zymosan by phagocytes in vivo, in the presence of absence of EDTA. MFI, mean fluorescence intensity (B) Representative fluorescent images of BAL-cells showing the uptake of FITC-labelled zymosan particles (green) in the presence or absence of EDTA. Nuclei are stained with DAPI (blue). (C) FSC and SSC flow cytometric analysis of unopsonised and BAL-opsonised C. albicans yeast. (D) Quantitation of neutrophil (CD11b+GR-1+) cells in the lungs of individual mice following intra-tracheal challenge with C. albicans in the presence or absence of EDTA. (TIF) [file pone.0035675.s002.tif]

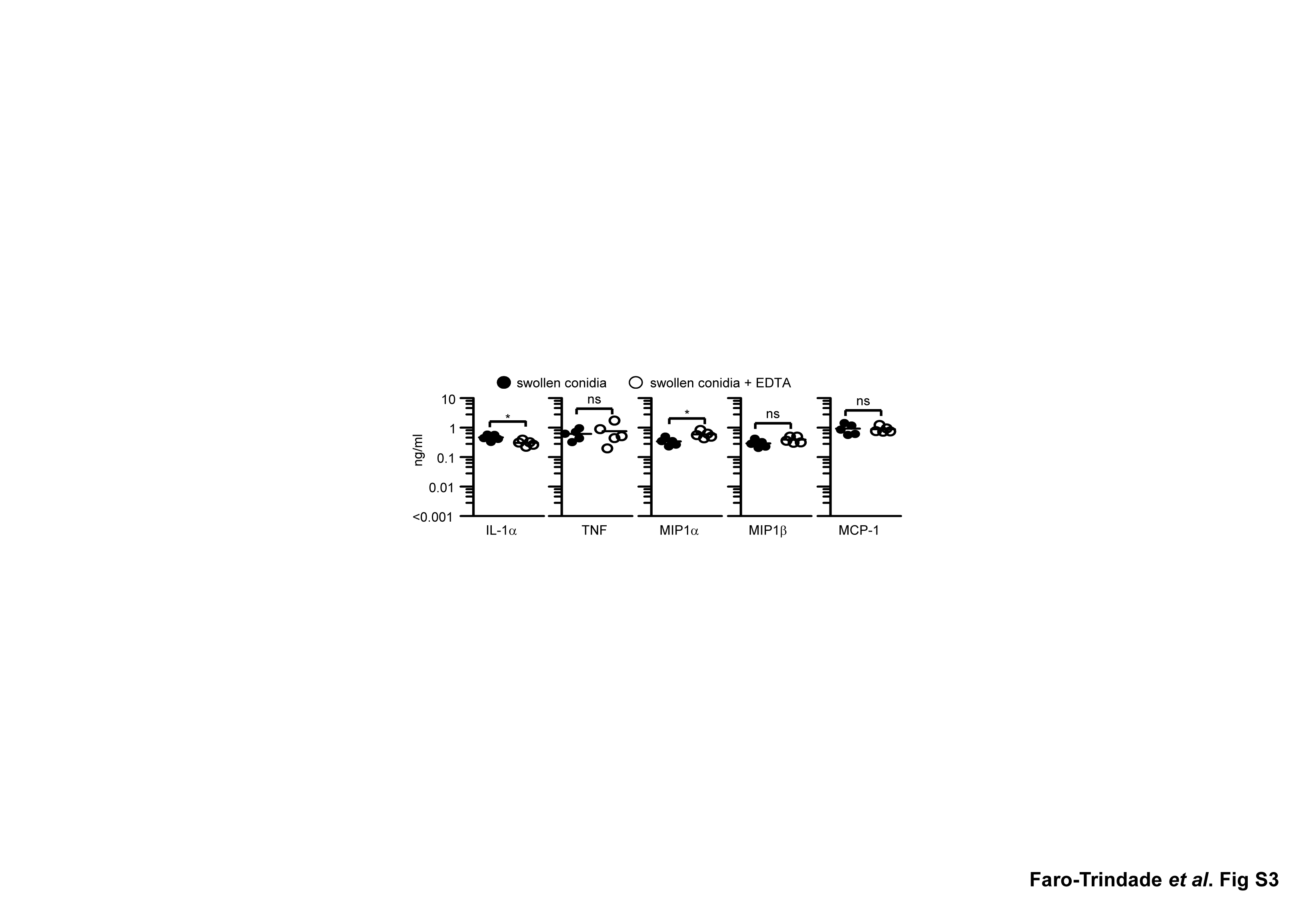

Supplement: Figure S3 — Surfactant does not influence pulmonary inflammatory response to Aspergillus swollen conidia. Production of selected cytokines and chemokines in the lungs of individual mice following intra-tracheal challenge with Aspergillus in the presence or absence of EDTA. (TIF) [file pone.0035675.s003.tif]

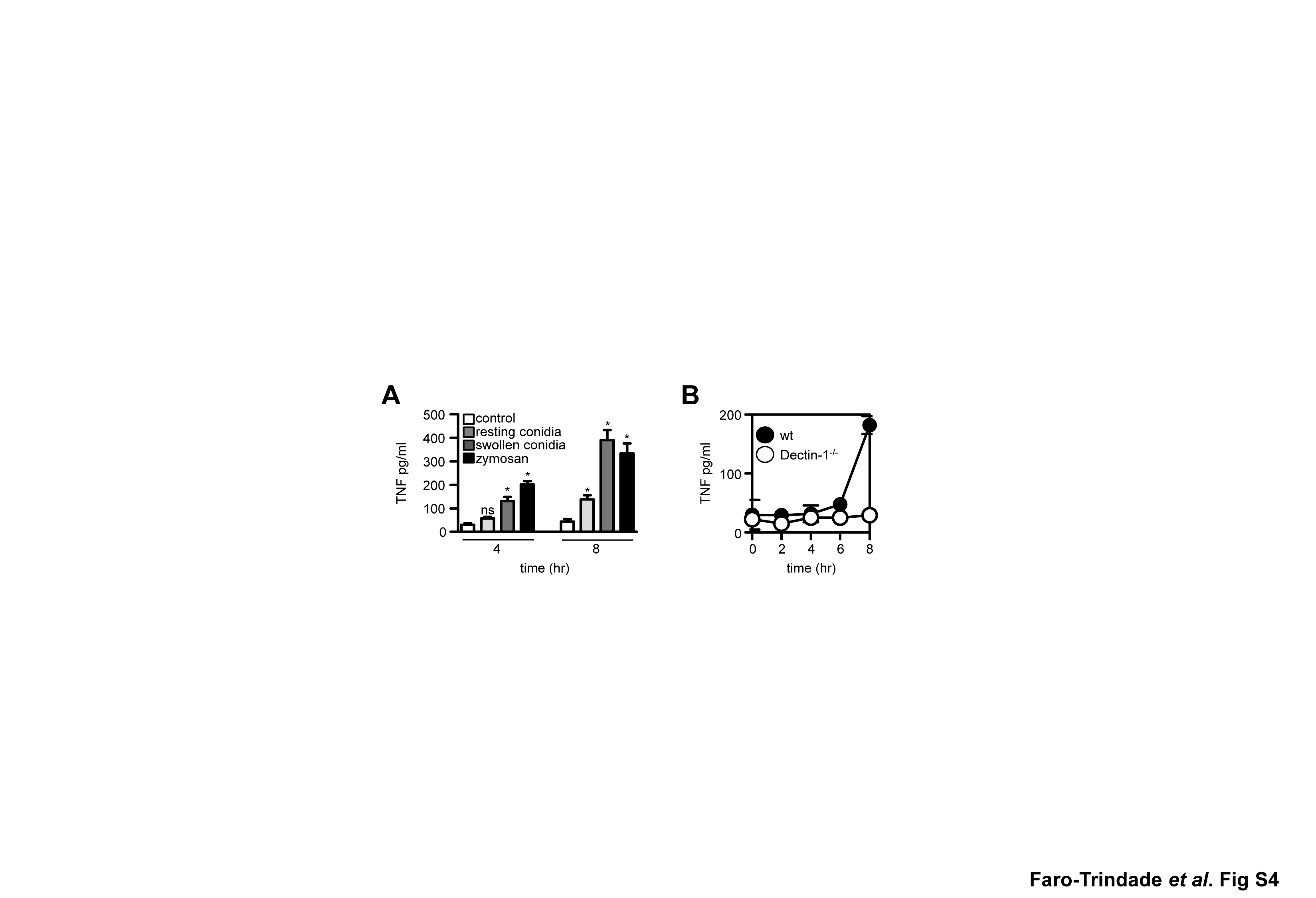

Supplement: Figure S4 — The induction of inflammatory responses to Aspergillus resting conidia is delayed and is Dectin-1 dependent. (A) The production of TNF at 4 and 8 hr following the infection of thioglycollate-elicited macrophages from wild-type mice with various particles, including swollen Aspergillus conidia, as indicated. Unstimulated cells are shown as a control. (B) The production of TNF over time following the infection of thioglycollate-elicited macrophages with resting Aspergillus conidia in wild-type or Dectin-1−/− mice, as indicated. The data shown are the mean ± SD, the wild type data is the same as shown in Figure 5 . (TIF) [file pone.0035675.s004.tif]

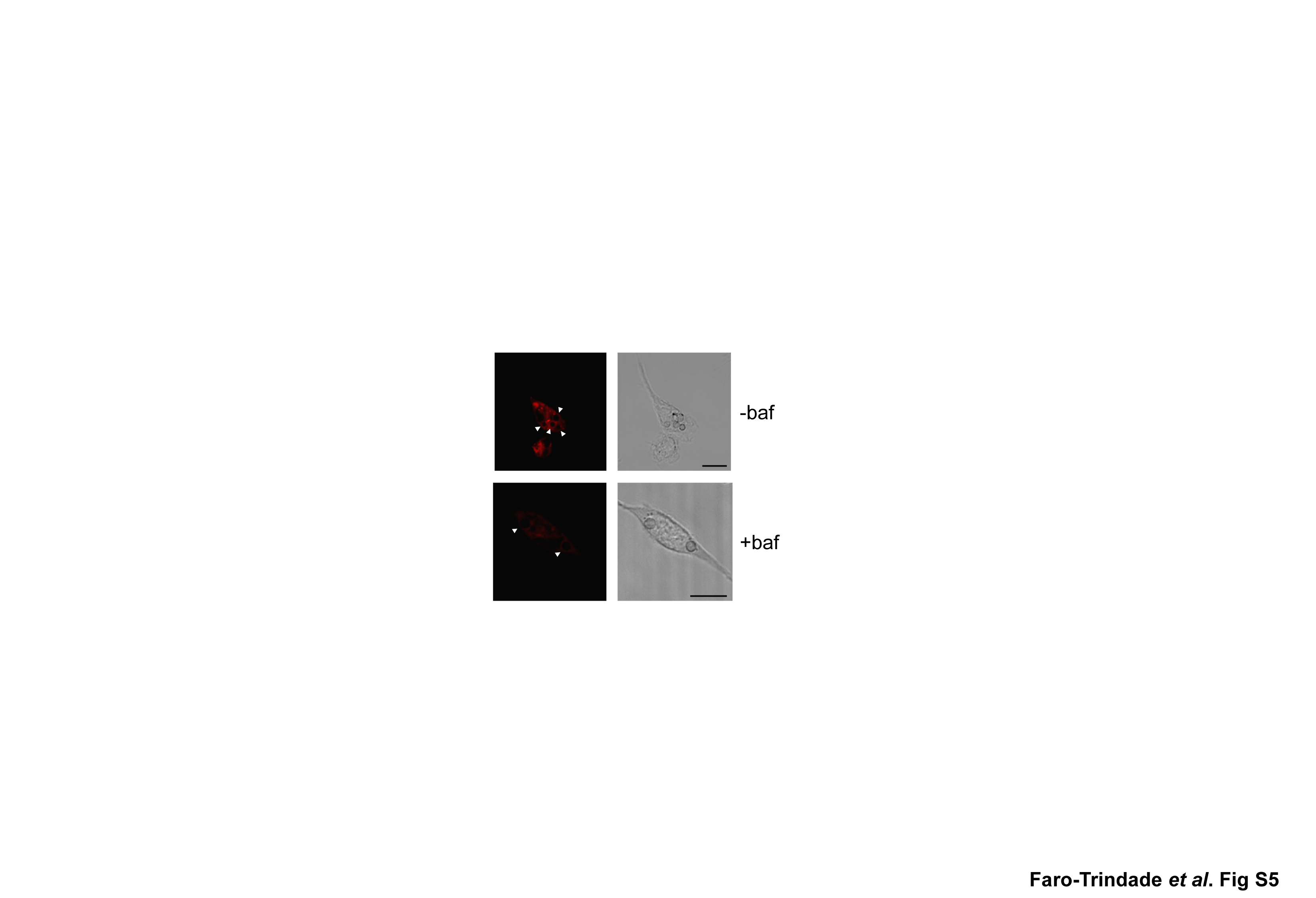

Supplement: Figure S5 — Acidification of conidial phagosomes can be inhibited by bafilomycin A1. Immunofluorescent confocal analysis of lysotracker red-labelled phagosomes at 6 hr after infection with and without bafilomycin A. Scale bar indicates 10 µm. (TIF) [file pone.0035675.s005.tif]
